# Transapical aortic valve replacement and concomitant coronary bypass grafting in on-pump beating fashion: a case report

**DOI:** 10.1093/jscr/rjaf119

**Published:** 2025-03-09

**Authors:** Shougo Takahashi, Kentaro Shirakura, Masahiro Tsutsui, Shingo Kunioka, Ryohei Ushioda, Yuya Kitani, Akiho Minoshima, Toshiharu Takeuchi, Naoki Nakagawa, Hiroyuki Kamiya

**Affiliations:** Department of Cardiac Surgery, Asahikawa Medical University, Midorigaoka Higashi 2-1-1-1, Asahikawa 078-8510, Japan; Department of Cardiac Surgery, Asahikawa Medical University, Midorigaoka Higashi 2-1-1-1, Asahikawa 078-8510, Japan; Department of Cardiac Surgery, Asahikawa Medical University, Midorigaoka Higashi 2-1-1-1, Asahikawa 078-8510, Japan; Department of Cardiac Surgery, Asahikawa Medical University, Midorigaoka Higashi 2-1-1-1, Asahikawa 078-8510, Japan; Department of Cardiac Surgery, Asahikawa Medical University, Midorigaoka Higashi 2-1-1-1, Asahikawa 078-8510, Japan; Department of Cardiology, Asahikawa Medical University, Midorigaoka Higashi 2-1-1-1, Asahikawa 078-8510, Japan; Department of Cardiology, Asahikawa Medical University, Midorigaoka Higashi 2-1-1-1, Asahikawa 078-8510, Japan; Department of Cardiology, Asahikawa Medical University, Midorigaoka Higashi 2-1-1-1, Asahikawa 078-8510, Japan; Department of Cardiology, Asahikawa Medical University, Midorigaoka Higashi 2-1-1-1, Asahikawa 078-8510, Japan; Department of Cardiac Surgery, Asahikawa Medical University, Midorigaoka Higashi 2-1-1-1, Asahikawa 078-8510, Japan

**Keywords:** transcatheter aortic valve replacement, coronary artery bypass grafting, on-pump

## Abstract

Herein, we report a case of transapical transcatheter aortic valve replacement and coronary artery bypass grafting performed in an on-pump beating fashion in an old woman with severe aortic valve stenosis, a porcelain aorta, severe calcified coronary artery disease, and a history of abdominal aortic replacement.

## Introduction

Transcatheter aortic valve replacement (TAVR) is widely used; however, the access route can be challenging in patients with difficult anatomic conditions. Additionally, the coexistence of severe coronary artery disease (CAD) that is unsuitable for percutaneous coronary intervention (PCI) complicates therapeutic options. Herein, we report a case of transapical TAVR and coronary artery bypass grafting (CABG) performed in an on-pump beating manner in an old woman with a porcelain aorta, severely calcified CAD, and a history of abdominal aortic replacement.

## Case report

**Figure 1:**
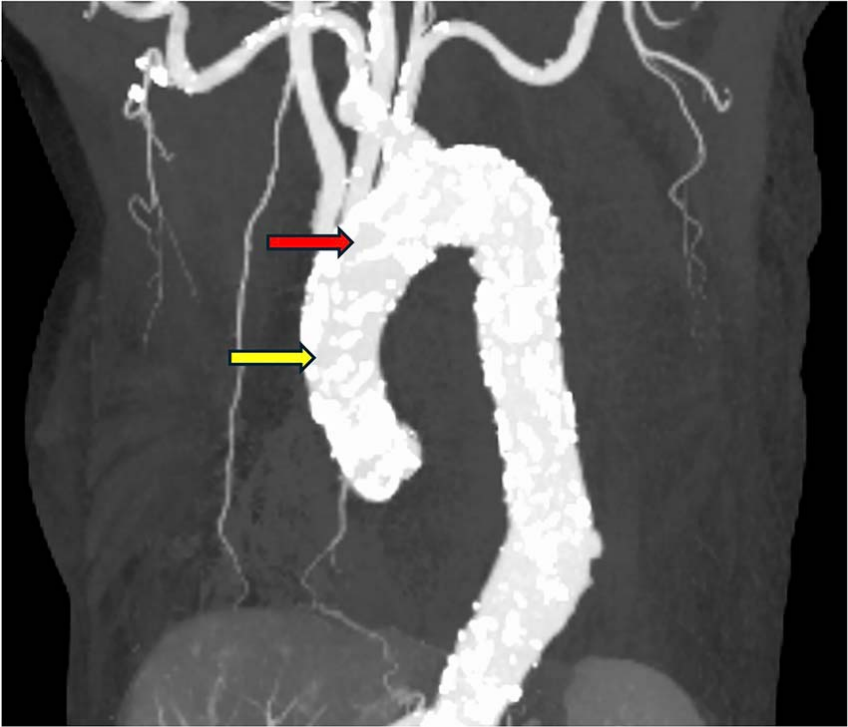
The ascending aorta is severely calcified. The red arrow indicates the arterial cannulation site, and the yellow arrow indicates the proximal anastomosis site of the vein graft.

**Figure 2:**
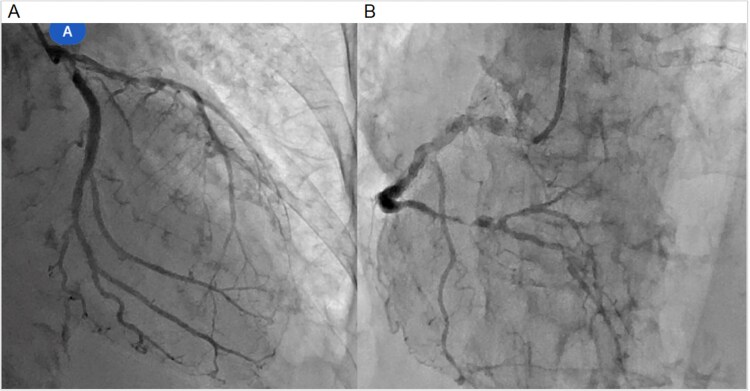
Coronary angiography: (A) left coronary artery and (B) right coronary artery.

**Figure 3:**
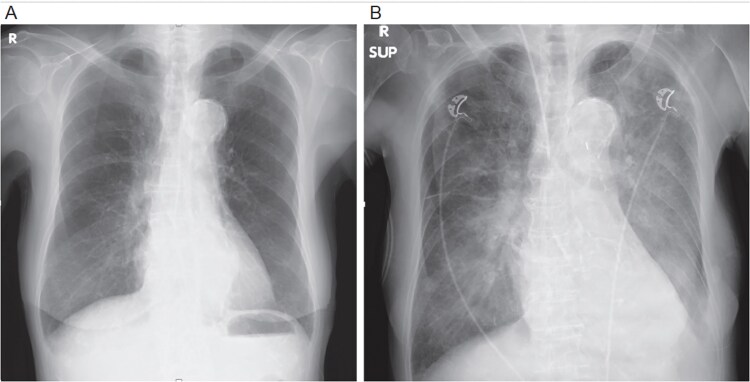
Chest X-ray: (A) immediately and (B) at the time of decompensation.

The patient is an 87-year-old woman. She was previously diagnosed with severe aortic valve stenosis by her general practitioner and was subsequently admitted to the cardiology department of the hospital for treatment. The patient had a preexisting medical history of angina pectoris, open repair of an abdominal aortic aneurysm, bilateral internal carotid artery stenosis, hypertension, chronic kidney disease, and type 2 diabetes mellitus. On admission, her vital signs were as follows: blood pressure, 123/40 mmHg; heart rate, 60 beats/min. The laboratory study conducted on admission revealed the following results: white blood cell counts 7600/μl, HbA1c 6.4%, C-reactive protein 0.69 mg/dl, and brain natriuretic peptide 1100 pg/ml. Echocardiography revealed a mean pressure gradient of 52 mmHg, maximum aortic flow velocity of 4.7 m/sec, and an aortic valve area (AVA) of 0.8 cm^2^. Aortic valve regurgitation was grade II. The left ventricular diastolic/systolic diameters were 49/30 mm, and ejection fraction was 67%. Contrast-enhanced computed tomography (CT) revealed calcifications and narrowing of the bilateral iliac arteries in addition to the porcelain aorta (Fig. 1). The patient had undergone percutaneous coronary intervention for angina pectoris 17 years previously. Coronary angiography (CAG) revealed severe stenosis in all three vessels (Figs 2 and 3), which was deemed unsuitable for PCI in the heart team discussion. Two days after admission, her condition worsened rapidly; she became catecholamine-dependent, and chest radiography revealed massive pulmonary edema (Fig. 3). An intra-aortic balloon pump (IABP) was placed via the right femoral artery approach, which is considered the preferred treatment option for severe AS because of the patient's advanced age and unstable hemodynamic condition necessitating IABP. However, the access route is highly limited, and TF-TAVAR is not a viable option following abdominal aortic replacement in the presence of severe iliac artery calcification. In addition, the ascending aorta is highly calcified, and there is a high risk of aortic dissection or stroke with catheterization when performing transaortic TAVR. Therefore, we decided to perform coronary artery bypass grafting and transapical TAVR (TA-TAVR) in an on-pump beating manner through medial sternotomy. Cardiopulmonary bypass (CPB) was performed with arterial cannulation of the aortic arch and venous cannulation of the right atrium. CABG was performed first: the left internal thoracic artery to the left anterior descending artery and the saphenous vein graft to the obtuse marginal artery and posterior descending artery for sequential grafting. Proximal anastomosis of the vein graft was performed on a calcification-free area of the ascending aorta using Heart-String III (Getinge, Sweden). TA-TAVR was performed using a 23 mm Sapien III (Enwards Life Sciences, Irvine, CA, USA). The patient underwent a re-thoracotomy for bleeding on the first postoperative day (POD). Thereafter, the patient recovered gradually, and the IABP was removed on the third POD, she was extubated on the fifth POD, and discharged from the intensive care unit on the eighth POD. The patient was transferred to a rehabilitation hospital on the 51st POD. One year after the surgery, the patient is now doing well.

## Discussion

According to current guidelines, surgical aortic valve replacement (SAVR) should be recommended even in older adults with severe AS and CAD requiring CABG [1, 2]. However, SAVR carries an extremely high risk in patients with severe frailty and/or calcification of the ascending aorta, which is unsuitable for aortic X-clamping, as in the present case. In such patients, simultaneous hybrid CABG and TAVR may be a reasonable option. Notably, there have only been a limited number of previous reports on this hybrid procedure. Honda *et al.* reported 27 cases of OPCAB and TAVR, including off-pump CABG (OPCAB) via median sternotomy + TF-TAVR in 14 patients, OPCAB via median sternotomy + transaortic-TAVR (TAo-TAVR) in six patients, minimally invasive cardiac surgery (MICS)-CABG via mini-left thoracotomy + TF-TAVI in six patients, and MICS-CABG + transsubclavian-TAVR in one patient [3]. One patient underwent aortic dissection due to proximal anastomosis of a vein graft onto the ascending aorta and subsequently underwent ascending aortic replacement; however, operative mortality was zero in their series. Baquero *et al.* reviewed 17 cases of transaortic TAVR and simultaneous off-pump CABG, including one of their own patients. The mean number of distal anastomoses was 1.8, and all patients survived [4]. Shimahara *et al.* reported on 49 patients who underwent OPCAB and TAVR via median sternotomy compared to 143 patients who underwent on-pump CABG and SAVR; postoperative outcomes were similar in both groups [5]. In their series, 36 patients underwent TAo-TAVI, and 13 underwent TF-TAVI. Thus, TAo-TAVR has been the preferred approach for TAVR in patients who underwent median sternotomy in previous studies. This seems reasonable, especially in patients with a healthy ascending aorta, because an additional skin incision and/or puncture site with a large sheath is required.

However, in the present case, the ascending aorta was highly calcified, and TAo-TAVR was considered impossible. Therefore, TA-TAVR was selected as the treatment modality for AS. In the early stages of TAVR, there were only two standard methods: TF and TA. With the development of devices, the percentage of TF-TAVR has increased and the emergence of alternative approaches, including TAo-, TSc-, TC-, and transcaval-TAVR, has reduced the frequency of TA-TAVR [6]. The mortality rate of TA-TAVR is higher than that of other approaches, and currently, many surgeons/cardiologists try to avoid TA-TAVR if possible [6]. Reflecting this trend, there are few reports on hybrid TA-TAVR and CABG. We found only one case series in which Mayr *et al.* reported 20 cases of successful hybrid OPCAB and TAVR, five of which underwent TA-TAVR [7]. In the present case, the hemodynamic condition was unstable, and the patient was suffering from ongoing severe heart failure; therefore, we performed hybrid CABG and TA-TAVR using CPB. In the literature, we could not find this combination, but CPB is the most reliable method of cardiopulmonary support, and we conclude that CPB would be useful in patients requiring CABG and TAVR, regardless of the approach modality.
